# Diseases of the Skin

**Published:** 1917-06

**Authors:** 


DISEASES OF THE SKIN. By Richard L. Sutton, M. D., Professor of Diseases of the Skin, University of Kansas School of Medicine; former Chairman of the Dermatological Section of the American Medical Association; member American Dermatological Association; Assistant Surgeon United States Navy, retired; Dermatologist to the Christian Church Hospital. Pp. 885; 693 illustrations and 8 colored plates. St. Louis: C. V. Mosby Company, 1916.

A work full of beautiful illustrations of the most important of the skin diseases. These pictures in themselves are a strong aid to diagnosis to one who has not had a large experience in this broad and difficult field. A large part of the text has been devoted to pathology and treatment. Obsolete therapeutic measures, and those that have been found ineffectual have been left out, or have been very briefly mentioned, and only those procedures that have proved of practical value in the author's experience have been dwelt upon. A striking virtue of the book is the simplicity and clearness of the presentation of a subject which, to say the least, is a bugbear to the average physician.

